# Anti-inflammatory action of cysteine derivative S-1-propenylcysteine by inducing MyD88 degradation

**DOI:** 10.1038/s41598-018-32431-0

**Published:** 2018-09-20

**Authors:** Jun-ichiro Suzuki, Yukihiro Kodera, Satomi Miki, Mitsuyasu Ushijima, Miyuki Takashima, Toshiaki Matsutomo, Naoaki Morihara

**Affiliations:** Central research laboratory, Wakunaga Pharmaceutical Co., Ltd., Hiroshima, Japan

## Abstract

The degradation of target proteins by small molecules utilizing the cellular proteolytic system is featured as a treatment strategy of several diseases. We found that *S*-1-propenylcysteine (S1PC) among several cysteine derivatives in aged garlic extract inhibited TLR-mediated IL-6 production by inducing the degradation of adaptor protein MyD88. We showed that S1PC directly denatured MyD88 and induced the formation of protein aggregates. Consequently, MyD88 was degraded by aggresome-autophagy pathway. On the other hand, *S*-allylcysteine, a structural analog of S1PC, failed to induce the degradation of MyD88 because of its inability to denature MyD88 although it also activated autophagy. Our findings suggest that S1PC induces MyD88 degradation through the denaturation of MyD88 and the activation of autophagy. Thus, S1PC may serve as the base to develop a therapeutic means for immune diseases associated with aberrant TLR signaling pathways.

## Introduction

Aged garlic extract (AGE) is manufactured by aging process in an aqueous ethanol solution for more than 10 months and has been shown to exert anti-inflammatory effects such as decreasing the production of TNFα and the expression of C-C motif chemokine ligand 2 (*Ccl2*) mRNA in animal models^1,2^. AGE contains various cysteine derivatives, that are responsible for its pharmacological effects^3–5^. *S*-1-Propenylcysteine (S1PC) and *S*-allylcysteine (SAC) that are similar in the structure are characteristic cysteine derivatives in AGE and are produced from γ-glutamyl-S1PC and γ-glutamyl-SAC, respectively in aging process^6–8^. Recently, we have shown that S1PC has an excellent bioavailability^9–11^ and enhances intestinal IgA production, and improves hypertension in spontaneously hypertensive rats (SHR)^12,13^.

Autophagy plays an important role in the degradation of protein aggregates and damaged organelles, and thus helps maintain protein quality control (PQC)^14–17^. Protein aggregates are post-translationally modified by the process such as ubiquitination, and then they are delivered to aggresome by binding to histone deacetylase 6 (HDAC6)^18,19^. Finally, they are degraded by the selective macroautophagy known as aggrephagy^3^. Several signaling molecules regulate the induction of macroautophagy. AMP-activated protein kinase (AMPK) is a major positive regulator and enhances the induction of autophagy by activating unc-51-like kinase 1/2 (Ulk1/2) under the starvation conditions. The activated ULK1 phosphorylates Beclin1-class III phosphatidylinositol 3-kinase (PI3KC3) complexes Atg9 that coordinately regulates the autophagy and membrane trafficking^20–24^. In addition, Atg5/7 is associated with the lipidation of microtubule-associated protein 1 A/1B-light chain 3 (LC3-I) and its conversion to LC3-II on the membrane of autophagosome during the process of autophagy^25–27^. The ubiquitin-modified proteins interact with LC3-II via autophagic receptor p62 on the membrane of autophagosomes fusing directly with lysosome^28^.

Toll-like receptor (TLR) signaling pathway is linked to innate immune responses and pathogenesis of inflammatory diseases^29–31^. TLRs recognize specific pathogen-associated molecular patterns (PAMPs) derived from microorganism and danger-associated molecular patterns (DAMPs) released from dead cells^29,32,33^. TLR dimerization induced by binding of its ligands recruits myeloid differentiation response protein 88 (MyD88), a common adaptor protein of TLRs, and IL-1 receptor-associated kinase 4 (IRAK4) to plasma membrane. This leads to the production of pro-inflammatory cytokines, including IL-6 and tumor necrosis factor-α (TNFα), through the activation of NF-κB^34–36^. MyD88-deficient mice fail to produce pro-inflammatory cytokines via TLR2 and TLR4, and show the decreased production of IL-1β and CCL2^37^.

Here we report that S1PC inhibited lipopolysaccharides (LPS)-induced TLR4 signaling pathway by inducing MyD88 degradation through its denaturation and the activation of autophagy.

## Results

### S1PC inhibited IL-6 production by blocking TLR signaling

**Figure 1:**
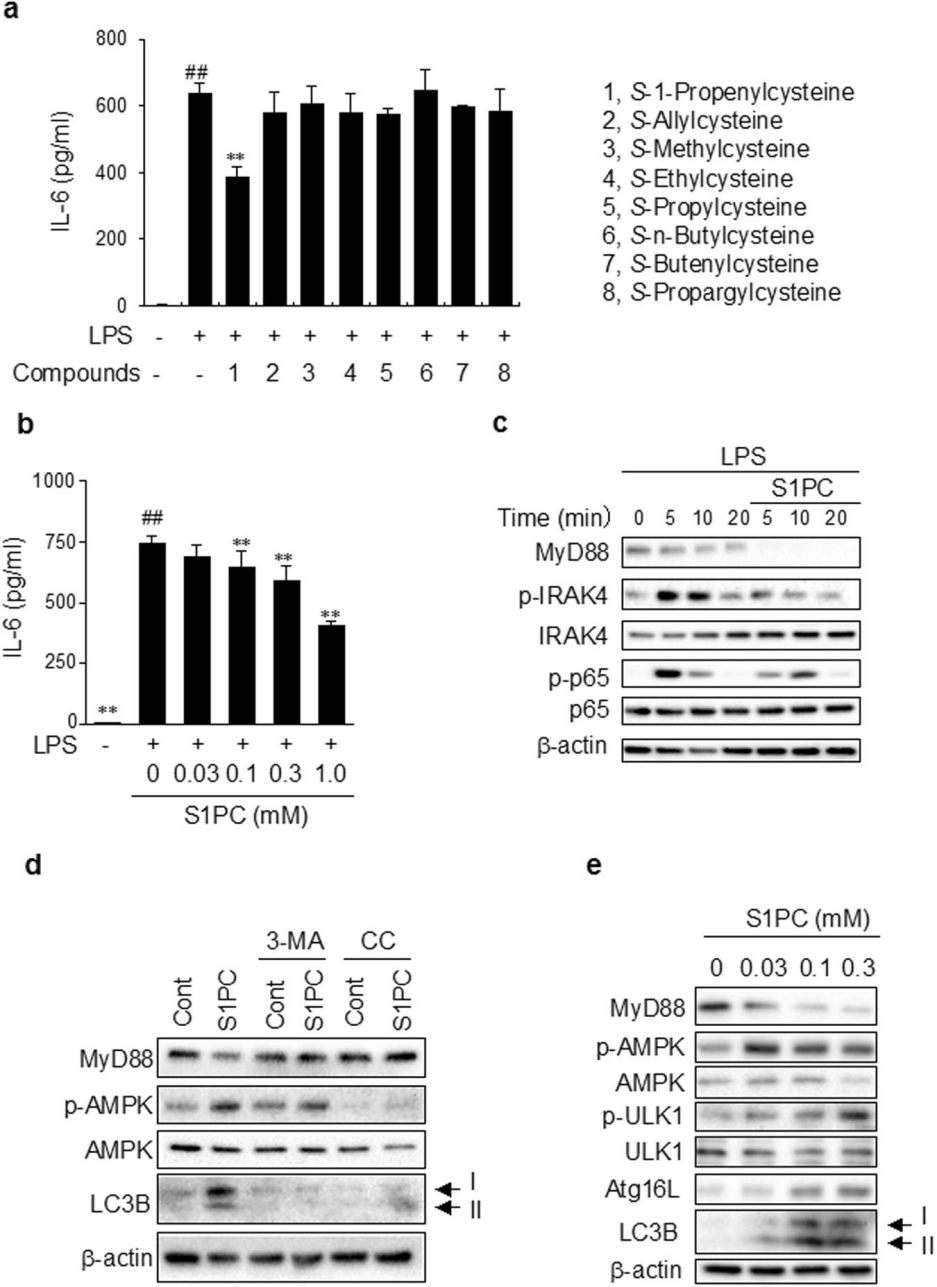
Effect of S1PC on TLR4 signaling pathway in splenic lymphocytes. (**a**) The effect of cysteine derivatives (0.3 mM) on IL-6 production induced by LPS (1 μg/ml) in splenic lymphocytes for 24 h were examined by ELISA. Data are shown as mean ± SD, n = 4-5. ** denotes significant difference (P < 0.01) compared to the treatment with LPS alone. (**b**) The effect of S1PC (0.03, 0.1, 0.3, and 1 mM) on IL-6 production induced by LPS (1 μg/ml) in splenic lymphocytes for 24 h was measured by ELISA. Data are shown as mean ± SD, n = 5-6. ** denotes significant differences (P < 0.01) compared to the treatment with LPS alone. (**c**) The effect of S1PC (0.3 mM) on TLR4 signaling pathway was examined by immunoblotting upon LPS stimulation (10 μg/ml) of splenic lymphocytes. Cell lysates were analyzed by western blotting with antibodies indicated. (**d**) Splenic lymphocytes were treated with or without 3-methyladenosine (3-MA; 1 mM) or compound C (CC; 10 μM) in the presence of S1PC (0.3 mM) for 10 min. Cell lysates were analyzed by western blotting with antibodies indicated. (**e**) The effect of S1PC (0.03, 0.1 and 0.3 mM) on autophagy-related signaling pathway was examined by immunoblotting in splenic lymphocytes. Cell lysates were analyzed by western blotting with the indicated antibodies.

We first investigated the effect of various cysteine derivatives on IL-6 production induced by a TLR4 agonist, LPS, in splenic lymphocytes (Fig. 1a). We found that S1PC inhibited LPS-induced IL-6 production in a concentration-dependent manner (Fig. 1b), whereas other compounds tested were ineffective. S1PC also suppressed IL-6 production induced by other TLR agonists such as TLR1/2 agonist, a triacylated lipopeptide (Pam3CSK4;), TLR5 agonist, a flagellin (FLA), TLR6/2 agonist, a lipopeptide (FSL-1) and TLR7 agonist, a single-strand RNA (ssRNA40/LyoVec) but not by TLR3 agonist, a double-strand RNA (poly(I:C)) (Supplementary Fig. 2). In addition, we found that S1PC did not affect cell viability and cell apoptosis at the concentrations tested (Supplementary Fig. 3a,b).

The effect of S1PC on TLR4 signaling pathway was shown in Fig. 1c. S1PC decreased the protein level of MyD88, whereas it had no effect on the level of *Myd88* mRNA (Supplementary Fig. S4). In addition, S1PC reduced the phosphorylation of IRAK4 and NF-κB p65, the downstream signaling molecules of MyD88, whereas it had no effect on the total protein levels of IRAK4 and NF-κB p65 (Fig. 1c).

To investigate whether MyD88 is degraded by autophagy-lysosome pathway, we examined the effect of 3-methyladenosine (3-MA), an inhibitor of PI3KC3, on S1PC-induced MyD88 degradation. As shown in Fig. 1d, 3-MA inhibited the decrease in MyD88 protein induced by S1PC. In addition, we found that compound C, an AMPK inhibitor, blocked S1PC-induced MyD88 degradation. These results suggested that S1PC induced MyD88 degradation by activating autophagy via AMPK.

We next examined the effect of S1PC on autophagy-related signaling molecules in splenic lymphocytes. As shown in Fig. 1e, S1PC increased the level of LC3B-I, LC3B-II and Atg16L, and enhanced the phosphorylations of ULK1 and AMPK, suggesting the activation of autophagy. On the other hand, SAC did not cause the reduction of MyD88 protein or the inhibition of IL-6 production although it also activated autophagy (Supplementary Fig. S5a,b). There was no difference in the cellular uptake of S1PC and SAC (Supplementary Fig. S6). The different effect of S1PC and SAC suggested that the induction of MyD88 degradation by S1PC required some other processes in addition to the activation of autophagy.

### S1PC induced MyD88 denaturation and post-translational modification

**Figure 2:**
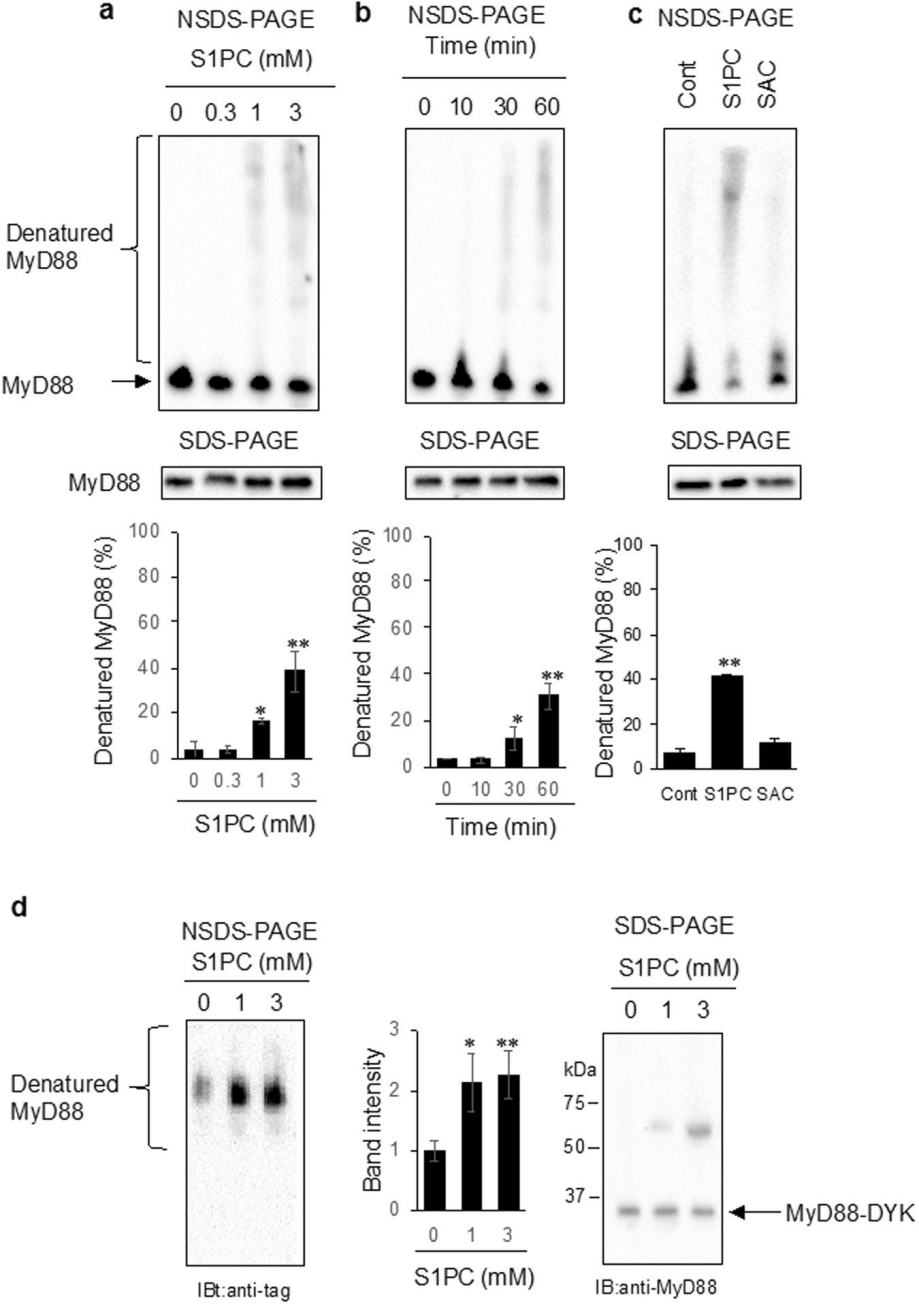
Effect of S1PC and SAC on the structure of MyD88 protein and lysine acetylation in splenic lymphocytes. The effect of S1PC and SAC on MyD88 protein was examined in cell lysates. Concentration-response relationship (0.3, 1 and 3 mM S1PC, 60 min) (**a**) and time-dependent change (3 mM S1PC, indicated times) (**b**) of the effect of S1PC and the comparison of the effect between S1PC and SAC (3 mM, 60 min) (**c**) on MyD88 protein were examined. Cell lysates treated with each compounds were analyzed by NSDS-PAGE and SDS-PAGE with anti-MyD88 antibody. Bar graphs show the percentage of the denatured MyD88 bands in the each immunoblotting. Data are shown as mean ± SD, n = 3. ** denotes significant differences (P < 0.01) compared to non-treated lysates. (**d**) The directly effect of S1PC on recombinant MyD88-DYK was examined. Recombinant MyD88-DYK treated with S1PC (1 and 3 mM, 60 min) was analyzed by NSDS-PAGE and SDS-PAGE with anti-DYKDDDDK tag and anti-MyD88 antibodies, respectively. Bar graphs show the relative of the band intensity in the each immunoblotting. Data are shown as mean ± SD, n = 3. ** denotes significant differences (P < 0.01) compared to non-treated solution.

Next, we examined the effect of S1PC and SAC on MyD88 protein using NSDS-PAGE method. As shown in Fig. 2a,b, the electrophoretic migration of MyD88 in S1PC-treated lysate was slower than that in untreated control. On the other hand, SAC treatment did not alter the migration pattern of MyD88. We also found that both S1PC and SAC treatment produced the same electrophoretic pattern when analyzed under the denaturing conditions by SDS-PAGE (Fig. 2c). Furthermore, it was found that the recombinant MyD88-DYK in S1PC-treated sample but not in untreated control sample was recognized by anti-DYKDDDDK tag antibody probably due to the conformation change of MyD88 (Fig. 2d). These results suggested that S1PC directly induced MyD88 denaturation.

### S1PC induced the formation of aggresome including MyD88 aggregates

**Figure 3:**
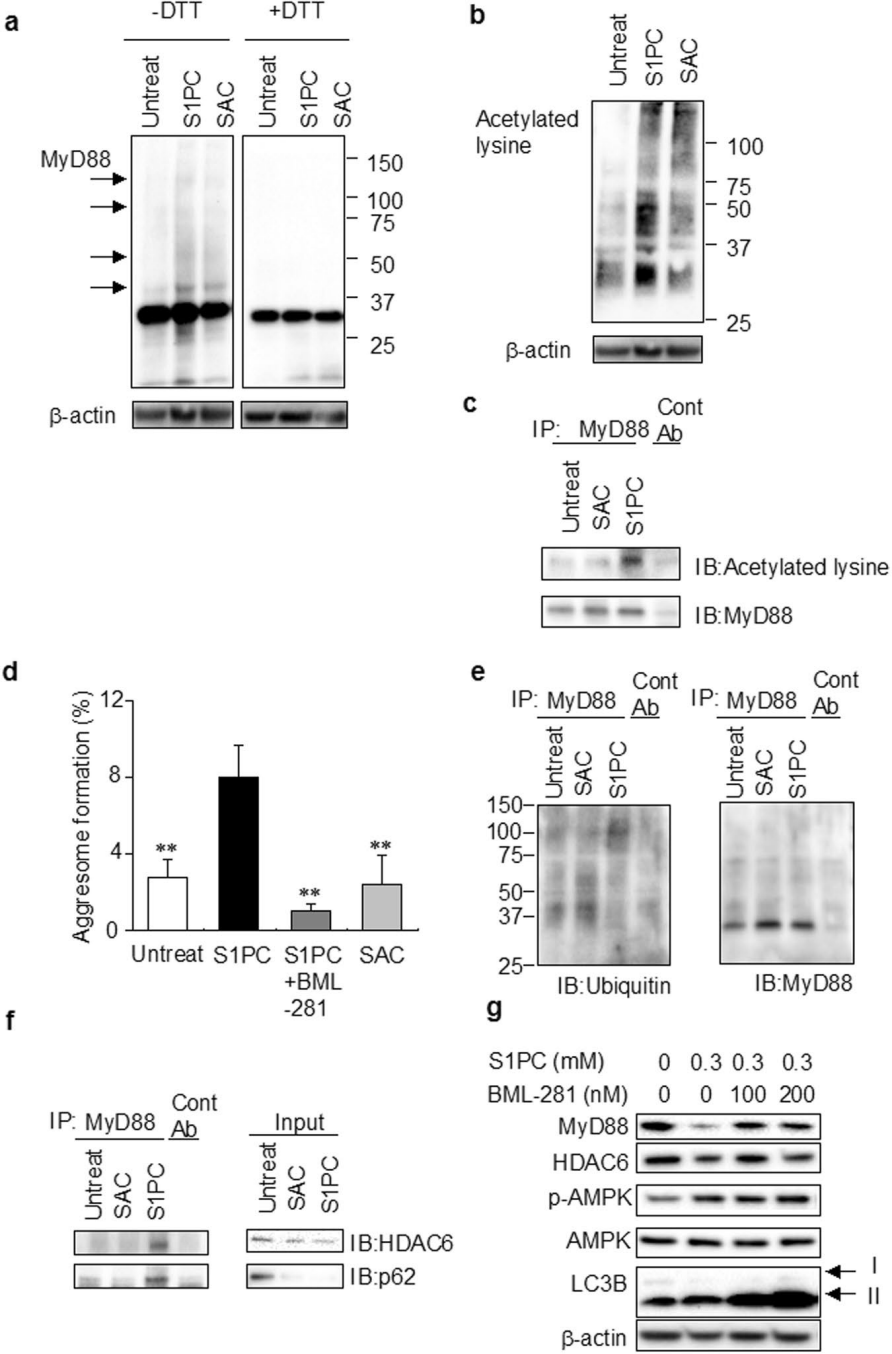
Effects of S1PC and SAC on aggresome formation. (**a**) The effect of S1PC (0.3 mM) and SAC (0.3 mM) on the disulfide bonds formation of MyD88 in splenic lymphocytes was measured by immunoblotting with anti-MyD88 antibody under the non-reducing condition (-DTT; left) and the reducing condition (+DTT; right). Black arrows show multiple bands of MyD88. (**b**) The effect of S1PC (0.3 mM) and SAC (0.3 mM) on lysine acetylation was examined by immunoblotting with anti-acetyl-lysine antibody under the non-reducing condition. (**c**) J774 cells were treated with S1PC (0.3 mM) and SAC (0.3 mM) for 10 min. Cell lysates were immunoprecipitated and analyzed by immunoblotting with antibodies indicated. (**d**) The effect of S1PC (0.3 mM) and SAC (0.3 mM) on aggresome formation with or without BML-281 (100 nM) in peritoneal macrophages was measured by aggresome detection kit and stained with DAPI for nuclei. Images were shown in Supplementary Fig. 7. The graph shows the percentage of aggresome forming cells. The percentage of aggresome formation was calculated as the number of aggresome-positive cells divided by the total number of DAPI-positive cells (>300 cells). Data are shown as mean ± SD, n = 3-4. ** denotes significant difference (P < 0.01) compared to S1PC treatment. (**e**,**f**) J774 cells were treated S1PC (0.3 mM) and SAC (0.3 mM) for 10 min. Cell lysates were immunoprecipitated and analyzed by immunoblotting with antibodies indicated. (**g**) Splenic lymphocytes were treated with or without BML-281 (100 and 200 nM) in the presence of S1PC (0.3 mM) for 10 min. Cell lysates were analyzed by immunoblotting with antibodies indicated.

The denaturation of protein triggers the formation of protein aggregates, which is mediated by disulfide bonds formation^38,39^. As shown in Fig. 3a, S1PC increased the multiple higher-molecular weight bands of MyD88 under the non-reducing conditions but these bands disappeared under the reducing conditions. On the other hands, the higher-molecular weight bands of MyD88 were not detectable in the SAC-treated lysate (Fig. 3a). We next examined the effect of S1PC and SAC on lysine acetylation that plays a crucial role in the regulation of protein aggregation^40^. As shown in Fig. 3b, S1PC increased the lysine acetylation of proteins, especially those having 30–50 kDa M.W., whereas SAC had little effect. In addition, we found that S1PC enhanced the lysine acetylation of MyD88 in murine macrophage cell line J774 cells, whereas SAC did not (Fig. 3c). These results suggested that S1PC promoted MyD88 aggregation mediated by lysine acetylation through the formation of disulfide bonds in MyD88 (Fig. 3a).

Protein aggregates are reported to be recruited to aggresomes, and then degraded by autophagy^18,28,41^. As shown in Fig. 3d, S1PC increased the formation of aggresome, whereas SAC did not. Generally, protein aggregates are polyubiquitinated and then interact with both p62 and HDAC6, that play an essential role in aggresome formation and transport of protein aggregates^18,19^. We thus examined whether HDAC6 was involved in S1PC-induced aggresome formation by using BML-281, a HDAC6 inhibitor, and found that it reduced the formation of aggresome by S1PC (Fig. 3d and Supplementary Fig. S7). As shown in Fig. 3e, higher-molecular weight bands were detected as polyubiquitinated MyD88 after S1PC treatment, whereas no such bands were formed by SAC treatment. In addition, we found that S1PC promoted the interaction of MyD88 with p62 and HDAC6 and reduced p62 and HDAC6 levels in total cell lysates, suggesting that they were degraded together with MyD88 (Fig. 3f). On the other hand, SAC induced the decrease only in p62, but not in HDAC6 (Fig. 3f). As shown in Fig. 3g, the inhibition of HDAC6 by BML-281 blocked S1PC-induced MyD88 degradation, whereas it did not suppress the phosphorylation of AMPK or the up-regulation of LC3B-II induced by S1PC. These results suggested that S1PC induced the degradation of MyD88 aggregates by activating two different pathways, HDAC6-dependent aggresome formation and HDAC6-independent autophagy-lysosome pathways.

### S1PC inhibited liver inflammation in SHR

**Figure 4:**
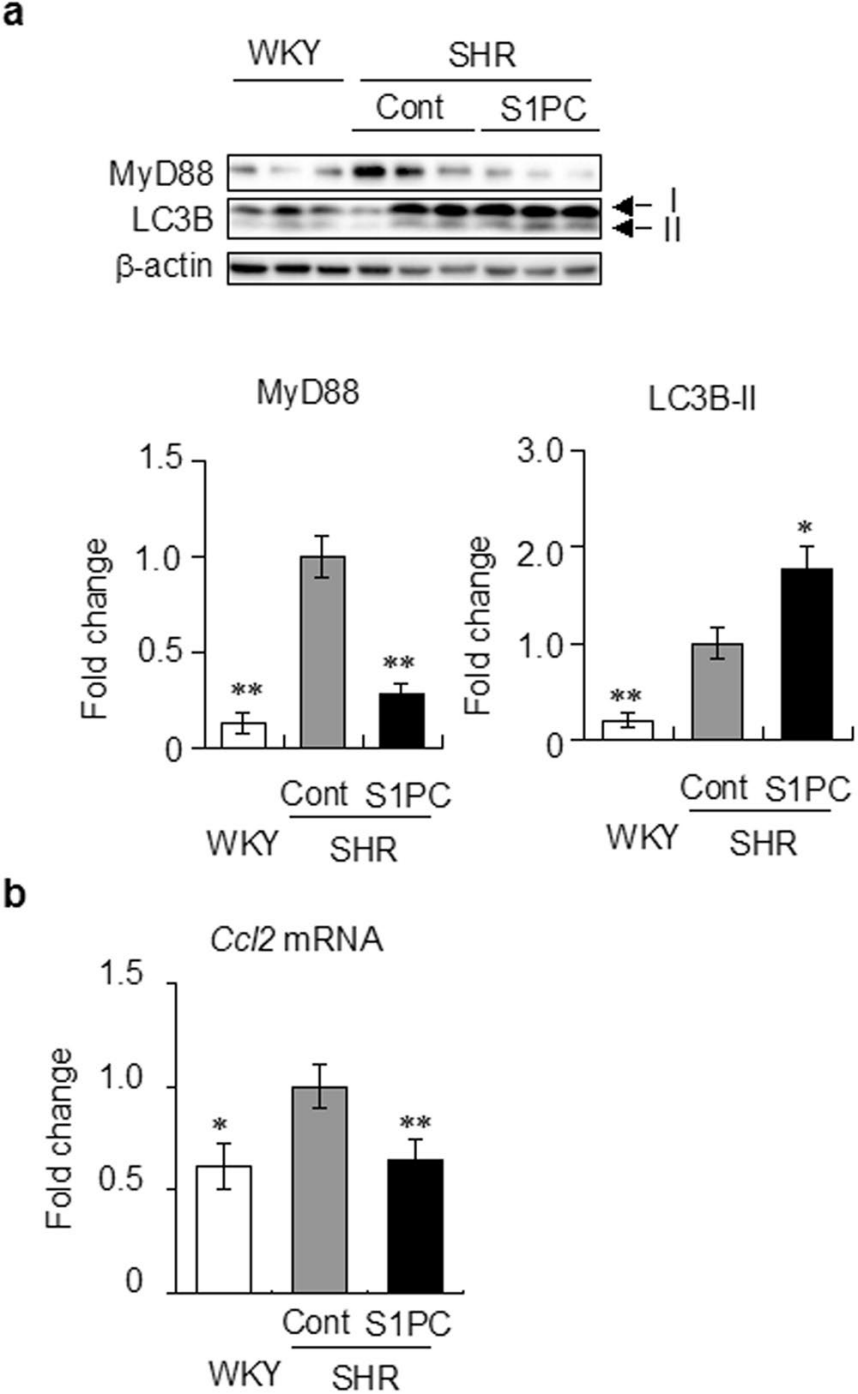
Effect of S1PC on the expression of MyD88 protein and *Ccl2* mRNA in liver of SHR. (**a**) S1PC (6.5 mg/kg) was orally administrated to SHR for 10 weeks. Liver lysates were analyzed by immunoblotting with antibodies indicated. β-actin is shown as loading control. Bar graphs show the quantification of protein level of MyD88 and LC3B-II. (**b**) The effect of S1PC on the expression of *Ccl2* mRNA in liver of SHR was examined by real-time PCR. Data are shown as mean ± SE, n = 5-6. * and ** denote significant differences (P < 0.05 and P < 0.01, respectively) compared with SHR control.

Our previous report showed that oral administration of S1PC significantly lowered the systolic blood pressure in SHR^13^. These rats exhibit chronic inflammation and the protein levels of MyD88 and LC3B-II in the liver of SHR were increased compared with that of normal WKY control rats (Fig. 4a). However, the levels of *Il6* and *Tnf* mRNA in liver were the same in both SHR and WKY rats (Supplementary Fig. S8). S1PC induced the degradation of MyD88 and increased the protein level of LC3B-II in SHR. In addition, we found that S1PC reduced the expression of pro-inflammatory chemokine *Ccl2* mRNA in the liver of SHR (Fig. 4b), suggesting that S1PC may help restrain the inflammatory response.

## Discussion

MyD88 is a key molecule in TLR signaling pathway linked to innate immune responses and inflammatory diseases^29–31,36^, and thus considered to be an important therapeutic target. However, no small molecule has been reported to induce the degradation of MyD88. The present study revealed that S1PC can induce MyD88 degradation through its combined action on denaturation of MyD88 and activation of autophagy. The degradation of MyD88 dampened the downstream TLR signaling pathway including the phophorylation of IRAK4 (Fig. 1c) and eventually led to the inhibition of IL-6 production (Fig. 1a,b). These changes induced by S1PC are similar to the phenotypic immune response of MyD88^−/−^ macrophages^42^, suggesting that S1PC is an effective anti-inflammatory agent. In addition, we found that S1PC inhibited the expression of *Ccl2* mRNA in SHR (Fig. 4b). The chemokine CCL2 plays an important role in the recruitment of monocytes into site of inflammation and thereby triggers several diseases including atherosclerosis, type 2 diabetes and rheumatoid arthritis^2,43,44^. The inhibition of CCL2 expression by S1PC may help ameliorate the inflammatory response by reducing monocyte recruitment. The present findings may serve as the basis for searching more potent compounds with the therapeutic potential.

We found that S1PC directly bound with MyD88 and induced its denaturation. The olefinic compounds such as S1PC have the potential to directly react with nucleophilic cysteine and lysine residues on protein surface^45,46^. These compounds are converted to thiols and then bound to cysteine residues by cross metathesis and Kirmse–Doyle ligation. Generally, the installation of thiols into proteins induces the chemical modifications that are similar to natural post-translational modifications (PTMs)^45–47^. In fact, allyl thiols derived from allyl compounds are considered as key metabolites that induce PTMs by selectively modifying cysteines over other nucleophilic residues such as lysine and histidine^45–47^. It is noteworthy that Toll/Interleukin-1 receptor (TIR) domain of MyD88 contains multiple cysteine and lysine residues and thus is susceptible to multiple modifications^48,49^. It is possible that S1PC binds to the TIR domain of MyD88. On the other hand, we found that SAC, a structural analog of S1PC failed to induce the degradation of MyD88 because of its inability to cause protein denaturation and lysine acetylation although it activated autophagy (Fig. 2e). Since the structures of S1PC and SAC differ only at the position of the double bond, it appears that this difference is important for induction of the degradation of MyD88. It is tempting to speculate that S1PC is converted to a thiol compound with high affinity for lysine residues and thus can induce the chemical modifications of MyD88, while SAC may not be.

Our results indicated that S1PC stimulated autophagy through AMPK activation (Fig. 1d). AMPK inactivates the mammalian target of rapamycin (mTOR) and directly activates ULK1. ULK1 induces the phosphorylations of Atg9 and Beclin-PI3KC3 complex^20–22^. In addition, we found that the S1PC-induced the activation of autophagy was inhibited by 3-MA and CC. These results suggested that S1PC induced the membrane nucleation through the activation of ULK1 and PI3KC3 by stimulating AMPK phosphorylation.

It was noted that the high concentration of S1PC (0.3 mM) was required for the inhibition of IL-6 production and the degradation of MyD88. Although the reason for this is not clear at this time, it may be due to the difficulty to maintain the effective intracellular concentration of S1PC that is influenced by its transport and metabolism. It is also conceivable that the high concentration of S1PC is required since it may need to interact with multiple cysteine and lysine residues in MyD88^48,49^. On the other hand, we found that the high concentrations of S1PC do not affect cell viability and causes no cell apoptosis, suggesting that S1PC induces MyD88 degradation by non-toxic action.

In conclusion, we showed that S1PC suppresses MyD88-dependent TLR signaling pathway in part through the induction of MyD88 degradation. This effect of S1PC is mediated by the induction of MyD88 denaturation and the activation of AMPK-associated autophagy. The present study may be useful for searching a therapeutic agent for inflammatory immune disease.

## Materials and Methods

### Preparation of S1PC and sulfur compounds

S1PC and SAC were prepared from AGE as previously described^12^. The chemical structures of compounds were determined by a LC-MS system consisting of an Ultimate 3000 and a Q-Exactive (Thermo Scientific, Waltham, MA, USA), and by a VNMRS-500 NMR spectrometer (VARIAN Inc., Palo Alto, CA, USA) at 500 MHz and 125 MHz. Cysteine derivatives were prepared according to previous report^6^.

#### *S*-Allylcysteine

^1^H–NMR (500 MHz, in D2O, δ): 3.00 (dd, 1 H, J = 7.5, 14.5 Hz, Ha), 3.10 (dd, 1 H, J = 4.5, 14.5 Hz, Hb), 3.25 (d, 1 H, J = 6.0 Hz), 3.94 (dd, 1 H, J = 4.5, 8.0 Hz), 5.24 (dd, J = 9.5,18.5, 1 H), 5.87 (m, 1 H);

^13^C–NMR (125 MHz, in D2O, δ): 30.63, 33.81, 53.44, 118.26, 133.44, 172.82, HRMS: observed [M + H]^+^  = 162.0577, calculated [M + H]^+^ = 162.0583.

#### *trans*-*S*-1-Propenylcysteine

^1^H–NMR (500 MHz, in D2O-NaOD, δ): 1.76 (d, 3 H, J = 7.0 Hz), 2.98 (dd, 1 H, J = 7.5, 14.5 Hz), 3.14 (dd, 1 H, J = 4.5, 14.5 Hz), 3.69 (dd, 1 H, J = 4.5, 7.5 Hz), 5.10–5.14 (m, 1 H), 6.02 (d, 1 H, J = 15.5 Hz).

^13^C–NMR (125 MHz, in D2O-NaOD, δ): 17.61, 33.53, 53.70, 119.92, 132.12, 172.73, HRMS: observed [M + H]^+^ = 162.0577, calculated [M + H]^+^ = 162.0583.

#### *cis*-*S*-1-Propenylcysteine

^1^H–NMR (500 MHz, in D2O, δ): 1.74 (d, 3 H, J = 7.0 Hz), 3.21 (dd, 1 H, J = 7.5, 15.0 Hz), 3.31 (dd, 1 H, J = 4.5, 15.0 Hz), 3.95 (dd, 1 H, J = 4.5, 7.5 Hz), 5.82–5.86 (m, 1 H), 6.01(d, 1 H, J = 9.5 Hz).

^13^C–NMR (125 MHz, in D2O-NaOD, δ): 13.89, 33.88, 54.16, 122.58, 127.78, 172.63. HRMS: observed [M + H]^+^ = 162.0577, calculated [M + H]^+ ^= 162.0583 The purity of S1PC and SAC was >97.0% and >99.0%, respectively.

### Antibodies and reagents

Anti-MyD88 Ab, anti-phospho IRAK4 Ab, anti-IRAK4 mAb, anti-phospho p65 Ab, anti-phospho ULK1 mAb, anti-ULK1 mAb, anti-phospho AMPKα mAb, anti-AMPKα mAb, anti-LC3B mAb and horseradish peroxidase (HRP)-conjugated Rabbit IgG were purchased from Cell Signaling Technology (Danvers, MA, USA). Anti-DYKDDDDK tag mAb, anti-tag antibody magnetic beads, DYKDDDDK peptide, coomassie G-250, compound C and HRP-conjugated anti-β-actin Ab were obtained from WAKO Pure Chemical Industries (Osaka, Japan). Lipopolysaccharide (LPS, *Salmonella enterica serotype typhimurium*) was purchased from Sigma Aldrich (St. Louis, MO, USA). Pam3CSK4, FLA-ST standard, FSL-1, ssRNA40/LyoVec and poly (I:C) HMW were obtained from InvivoGen (San Diego, CA, USA). Halt protease & phosphatase inhibitor single-use cocktail was purchased from Thermo Scientific. RIPA lysis buffer was obtained from Merck Millipore (Billerica, MA, USA). 3-methyladenine was purchased from Santa cruz Biotechnology (Dallas, TX, USA).

### Cell culture and treatment

Splenic lymphocytes were isolated as previously described^23^. For *in vitro* IL-6 production assay, splenic lymphocytes were plated at 1 × 10^6^ cells per well in 48-well plate (Corning, NY, USA). The cells were cultured in RPMI-1640 containing 10% FBS, penicillin (100 U/ml), and streptomycin (100 μg/ml) with or without tested compounds in the presence or absence of LPS (1 μg/ml) at 37 °C for 24 h. After cultivation, the supernatants were collected. IL-6 level in the supernatant was measured using Mouse IL-6 ready-set-go ELISA set (Thermo Scientific), and then cell viability was measured using Cell counting kit-8 (Dojindo, Kumamoto, Japan) and cell apoptosis was assessed using Annexin V-FITC Apoptosis Detection Kit (Thermo Scientific). For aggresome formation assay, peritoneal macrophages were obtained from female C57/BL6N mice (7–10 wks) and incubated with culture medium at 37 °C for 3 h. Non-adherent cells were isolated and then cultured in medium with 20 ng/ml M-CSF. After 72 h, cells were treated with S1PC (0.3 mM) and SAC (0.3 mM) in the presence or absence of 100 nM BML-281 (Enzo Life Sciences, NY, USA) and aggresomes were detected using aggresome detection kit (Enzo Life Sciences). The stained cells were analyzed using BZ-9000 (KEYENCE, Osaka, Japan). The percentage of aggresome formation was calculated as the number of aggresome-positive cells divided by the total number of DAPI-positive cells.

Human kidney cell line 293FT cell and murine macrophage cell line J774 were maintained in DMEM containing 10% FBS, penicillin (100 U/ml) and streptomycin (100 μg/ml).

### Mass spectrometry analysis

Splenic lymphocytes (1 × 10^7^ cells/ml) were pretreated with RPMI1640 containing 1% FBS for 30 min. Subsequently, the cells were treated with S1PC (0.3 mM) or SAC (0.3 mM) at 37 °C for 5 min. The cells were washed three times with cold PBS, suspended in the mixture of acetonitrile and water (60:40), and centrifuged at 19,000 g at 4 °C for 10 min. LC-MS analysis was performed as previously described^24^. LC separation was carried out on a Hypersil GOLD C18 column (150 × 2.1 mm, 1.9 μm, Thermo Scientific) equipped with a Hypersil GOLD C18 guard column at 40 °C. The high-resolution, accurate-mass analysis was performed with an electrospray ionization (ESI) source in targeted SIM mode (*m/z* 162.0583 as [M + H]^+^ ions of S1PC and SAC). Calibration curves were prepared over a range of 6–60 μM in plasma.

### Treatment with compounds and western Blot analysis

Splenic lymphocytes (1 × 10^7^ cells/ml) were pretreated with RPMI1640 containing 1% FBS in the presence or absence of 3-methyladenosine (3-MA; 1 mM), compound C (CC; 10 μM) and HDAC6 specific inhibitor (BML-281; 100 and 200 nM) for 30 min. Subsequently, the cells were treated with S1PC (0.03, 0.01 and 0.3 mM) or SAC (0.03, 0.01 and 0.3 mM) in the presence or absence of 10 μg/ml LPS at 37 °C for 2–20 min. Western blot analysis was performed as previously described^23^. Immunoreactions were performed using an ECL Prime western blotting detection system (GE Healthcare, Little Chalfont, Buckinghamshire, England) and SuperSignal™ West Femto Maximum Sensitivity Substrate (Thermo Scientific) and visualized bands were analyzed on a V3 Western Workflow using Image Lab^TM^ software (BioRad, Hercules, CA, USA).

### Preparation of cell lysates and recombinant MyD88-DYK

J774 cells were lysed with deionized water containing protease and phosphatase inhibitor and sonicated using ultrasonic homogenizer (TAITEC, Saitama, Japan). The lysates were centrifuged at 10,000 g for 10 min at 4 °C. The supernatants were collected. Cell lysates were treated with S1PC (0.3, 1 and 3 mM) or SAC (3 mM) for 10-60 min at 37 °C. 293FT cells were transfected with pcDNA3.1+/c-(k) dyk containing MyD88 (Genscript, NJ, USA). After 24 h, cells were lysed with deionized water containing protease and phosphatase inhibitor and sonicated using ultrasonic homogenizer. Recombinant MyD88-DYK was purified using anti-DYKDDDDK tag magnetic beads. Recombinant MyD88-DYK was treated with S1PC (1 and 3 mM) for 60 min at 37 °C.

### NSDS-PAGE (Native SDS-PAGE)

Protein samples were added to 2.5 μl of 5X NSDS sample buffer (100 mM Tris HCl, 30% (v/v) glycerol, 1.0% (w/v) Coomassie G-250). Samples were separated using 4–15% SDS-PAGE gradient gel in running buffer (100 mM Tris HCl, 0.03% (w/v) SDS) at 4 °C and immunoblotted with indicated antibodies.

### Immunoprecipitation

J774 cells were treated with S1PC (0.3 mM) or SAC (0.3 mM) at 37 °C for 10 min and lysed using a lysis buffer. Lysates were immunoprecipitated using anti-MyD88 mAb and protein G magnetic beads (Merck Millipore). The immune complexes were washed four times with lysis buffer, boiled in SDS sample buffer, and subjected to western blot analysis with the indicated antibodies.

### Animal care, treatment and sample preparation

Male Wistar Kyoto rats (WKY/Izm) and SHR/Izm (9 wks) were purchased from Japan SLC (Shizuoka, Japan). Female C57BL/6 N mice (6–10 wks) were obtained from CLEA Japan (Tokyo, Japan). Animals were individually housed under specific pathogen-free condition. They were provided a commercial diet (CE-2, CLEA Japan) and water *ad libitum* under a 12 h-light-dark cycle, controlled temperature (22 ± 1 °C) and humidity (50 ± 5%). SHR was orally administered distilled water or S1PC (6.5 mg/kg BW) for 10 wks. Rat livers were homogenized using the Multi-Beads shocker (YASUI KIKAI, Osaka, Japan) under the frozen condition by liquid nitrogen. *In vivo* studies had been approved by the Animal Care and Use Committee of Wakunaga Pharmaceutical Co., Ltd (approval number: 170 and 203). This investigation conforms with the Guide for the Care and Use of Laboratory Animals published by the US National Institute of Health (NIH Publication, 8^th^ Edition, 2011).

### Real-time PCR analysis

Total RNA was isolated from cells and tissues using TRIzol reagent. cDNA was prepared from total RNA using a PrimeScript RT reagent kit with gDNA Eraser (Takara, Shiga, Japan). The quantitative real-time PCR reactions were performed using SYBR Premix Ex Taq II (Takara) to determine relative expression levels of target genes to 18S in a Piko Real Real-time PCR system (Life technologies, Carlsbad, CA, USA). The specific mRNA levels were calculated using the comparative CT (ΔΔCT) method. The following primers:

forward rat *Ccl2* 5′-TGGGCCTGTTGTTCACAGTT-3′; reverse rat *Ccl2* 5′-ACCTGCTGCTGGTGATTCTC; forward mouse *Myd88* 5′-ACCACCCTTGATGACCCCCT-3′; reverse mouse *Myd88 5*′*-*GTCACGGTCGGACACACACA-3′; forward mouse/rat *Rn18s* 5′-ATGCGGCGGCGTTATTCC-3′; reverse mouse/rat *Rn18s* 5′-ATCTGTCAATCCTGTCCGTGTC-3′

### Statistical analysis

After outlier values were rejected using the Tomson’s rejection test in all data, analysis of variance (ANOVA) followed by Bonferroni’s multiple comparison tests were applied to identify statistically significant difference between the test and control groups using a STATISTICA software package (Dell software). Differences at P-Values less than 0.05 were considered to be statistically significant.

## Electronic supplementary material

Supplementary information
