# Supplementary material for: Anti-inflammatory action of cysteine derivative S-1-propenylcysteine by inducing MyD88 degradation
Source: Sci Rep. 2018 Sep 20;8:14148. doi: 10.1038/s41598-018-32431-0 (PMC6148218; doi:10.1038/s41598-018-32431-0)
Supplement: Supplementary file 1 — Supplementary information [file 41598_2018_32431_MOESM1_ESM.pdf]

Anti-inflammatory action of cysteine derivative S-1-propenylcysteine by inducing MyD88 degradation

*Jun-ichiro Suzuki, Yukihiro Kodera, Satomi Miki, Mitsuyasu Ushijima,
Miyuki Takashima, Toshiaki Matsutomo and Naoaki Morihara

Central research laboratory, Wakunaga Pharmaceutical Co., Ltd

Corresponding Author's information:

Jun-ichiro Suzuki

Central research laboratory, Wakunaga Pharmaceutical Co., Ltd.

Address: 1624 Shimokotachi, Koda-cho, Akitakata-shi, Hiroshima 739-1195, Japan

E-mail address: suzuki_j@wakunaga.co.jp

TEL: +81-826-45-2331

FAX: +81-826-45-4351

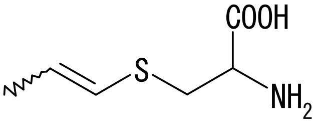

S-1-Propenylcysteine (S1PC)
(**1**)

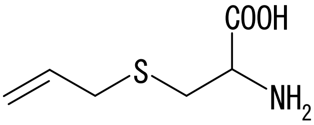

S-Allylcysteine (SAC)
(**2**)

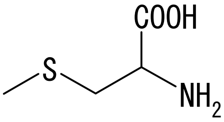

S-Methylcysteine
(**3**)

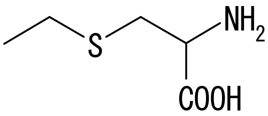

S-Ethylcysteine
(**4**)

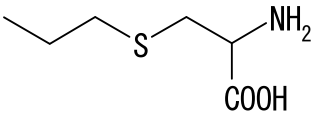

S-Propylcysteine
(**5**)

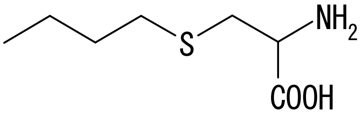

S-n-Butylcysteine
(**6**)

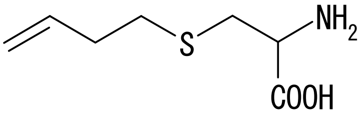

S-Butenylcysteine
(**7**)

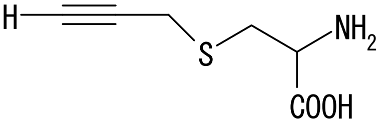

S-Propargylcysteine
(**8**)

Fig. S1 Structures of cysteine derivatives

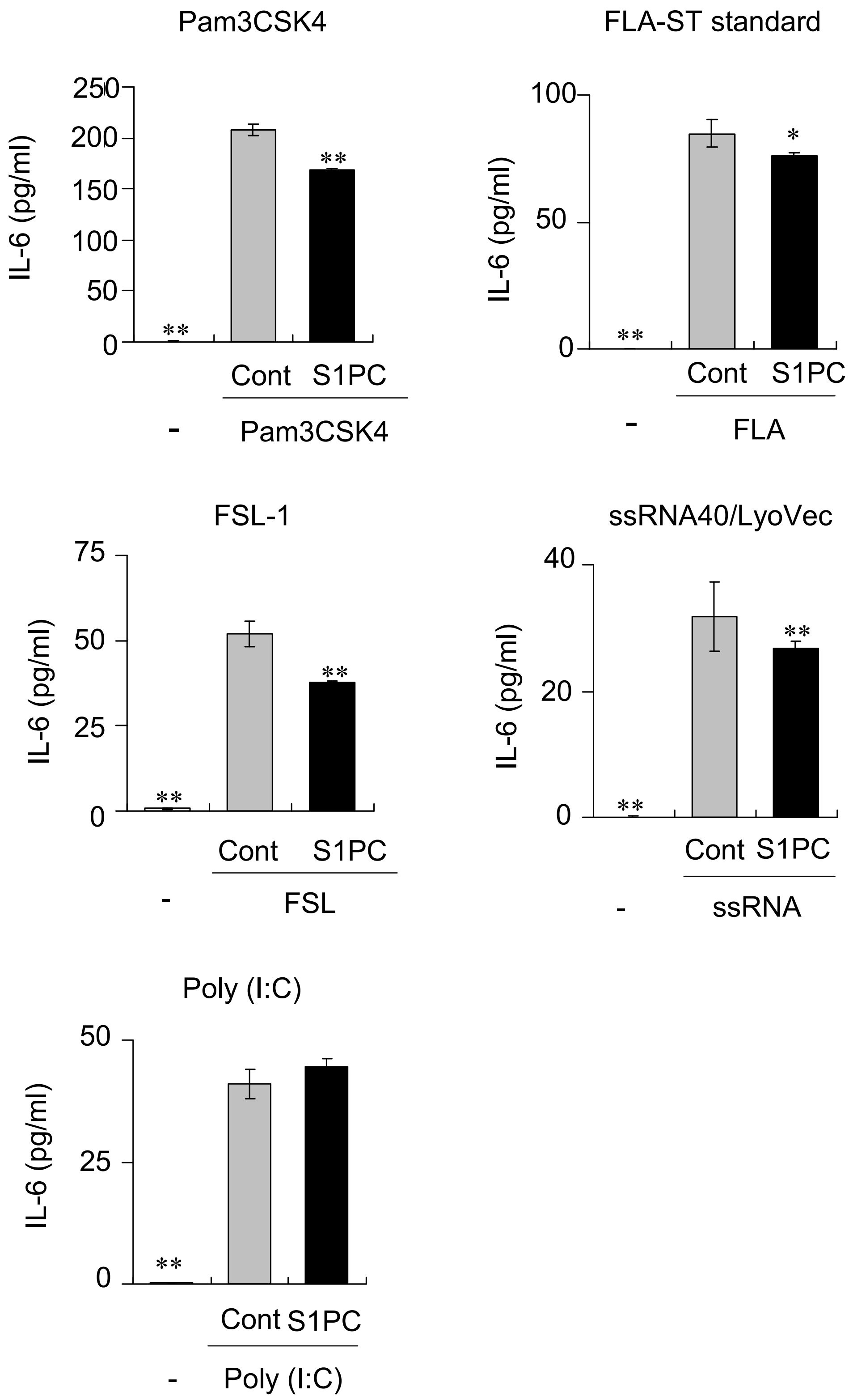

Fig. S2. Effect of S1PC on Il-6 production induced by TLR agonists in splenic lymphocytes. The effect of S1PC (0.3 mM) on IL-6 production induced by Pam3CSK4 (1 μg/ml), FLA-ST standard (10 μg/ml), FSL-1 (1 μg/ml), ssRNA40/LyoVec (10 μg/ml) and Poly (I:C) (10 μg/ml) in splenic lymphocytes for 24 h was measured by ELISA. Data are shown as mean ± SD, n=4-5. * and ** denote significant differences ($P<0.05$ and $P<0.01$, respectively) compared to the treatment with the TLR agonist alone.

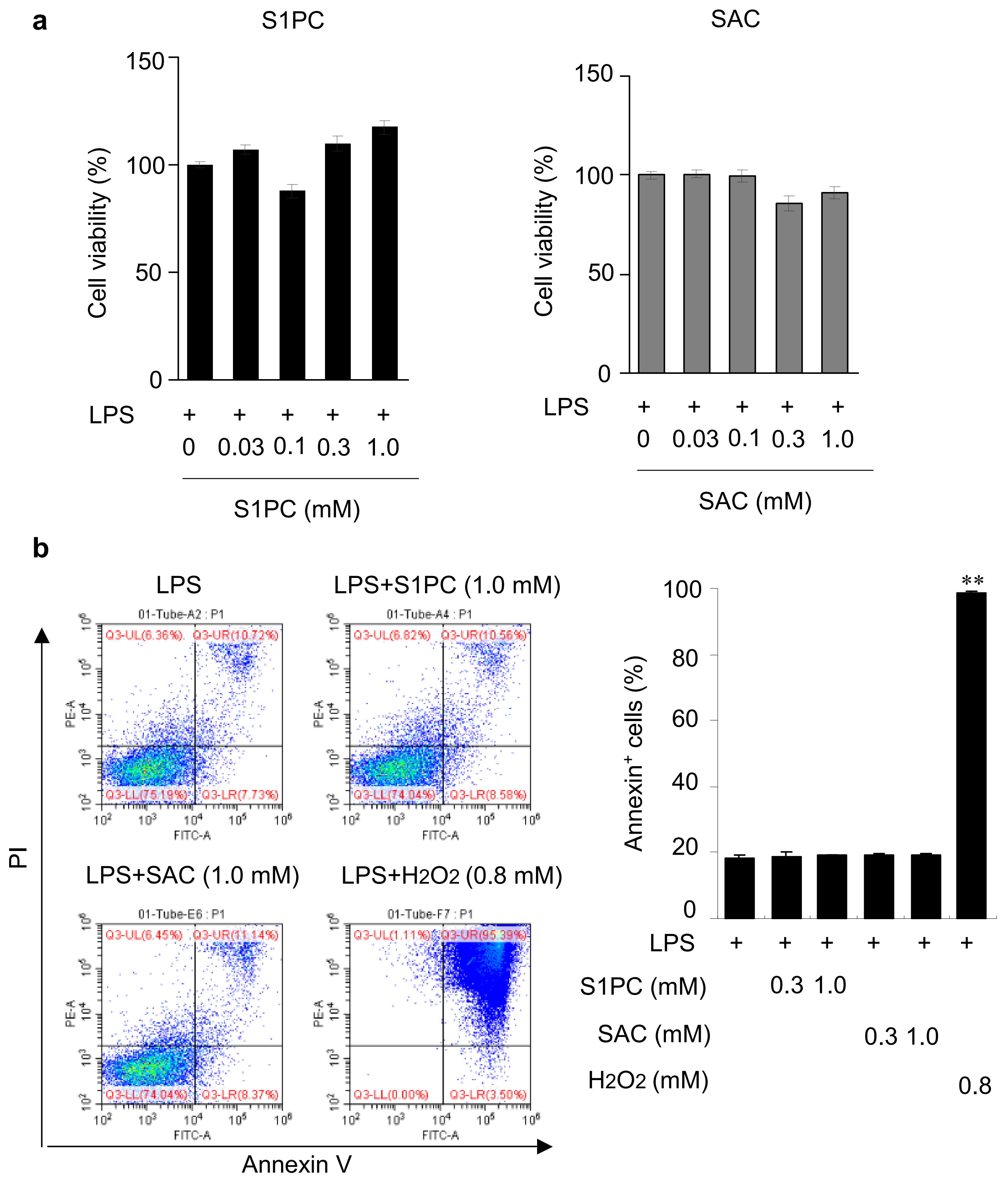

Fig. S3. Effect of S1PC and SAC on cell viability in splenic lymphocytes.
(a) Viability of splenic lymphocytes treated S1PC (0.03, 0.1, 0.3, and 1.0 mM) or SAC (0.03, 0.1, 0.3 and 1.0 mM) in the presence of LPS (1 μg/ml) for 24 h was examined by cell counting kit-8. Data are shown as mean ± SD, n=4-5. (b) Effects of S1PC and SAC on cell apoptosis in splenic lymphocytes. Splenic lymphocytes treated S1PC (0.3, and 1.0 mM) , SAC (0.3 and 1.0 mM) or H_2O_2 (0.8 mM) in the presence of LPS (1 μg/ml) for 24 h was examined by Annexin V-FITC Apoptosis Detection Kit. Data are shown as mean ± SD, n=4. ** denotes significant differences (P<0.01) compared to the treatment with LPS alone.

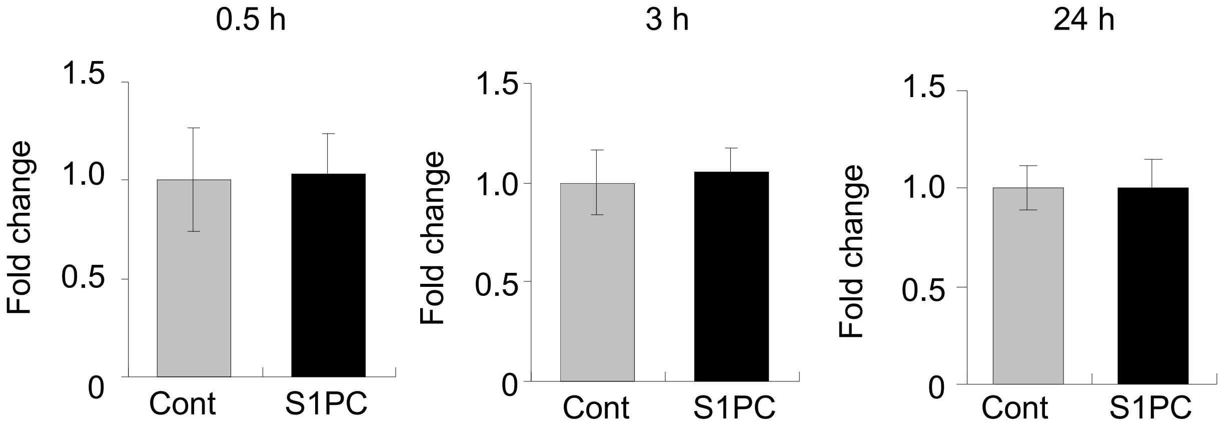

Fig. S4. Effect of S1PC on the expression of *Myd88* mRNA in splenic lymphocytes.
The effect of S1PC (0.3 mM) on the expression of *Myd88* mRNA for 0.5, 3 and 24 h in splenic lymphocytes was examined by real-time PCR. Data are shown as mean ± SD, n=3-4.

a

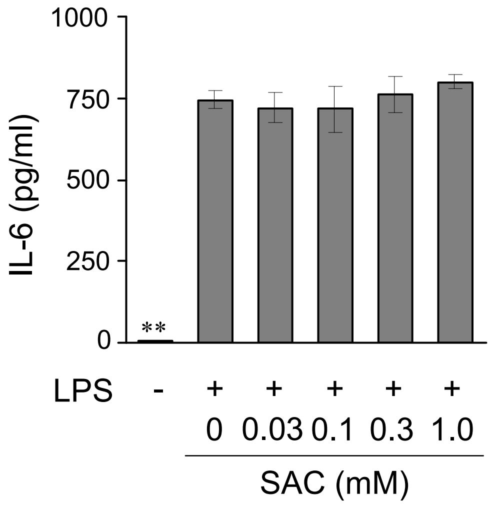

b

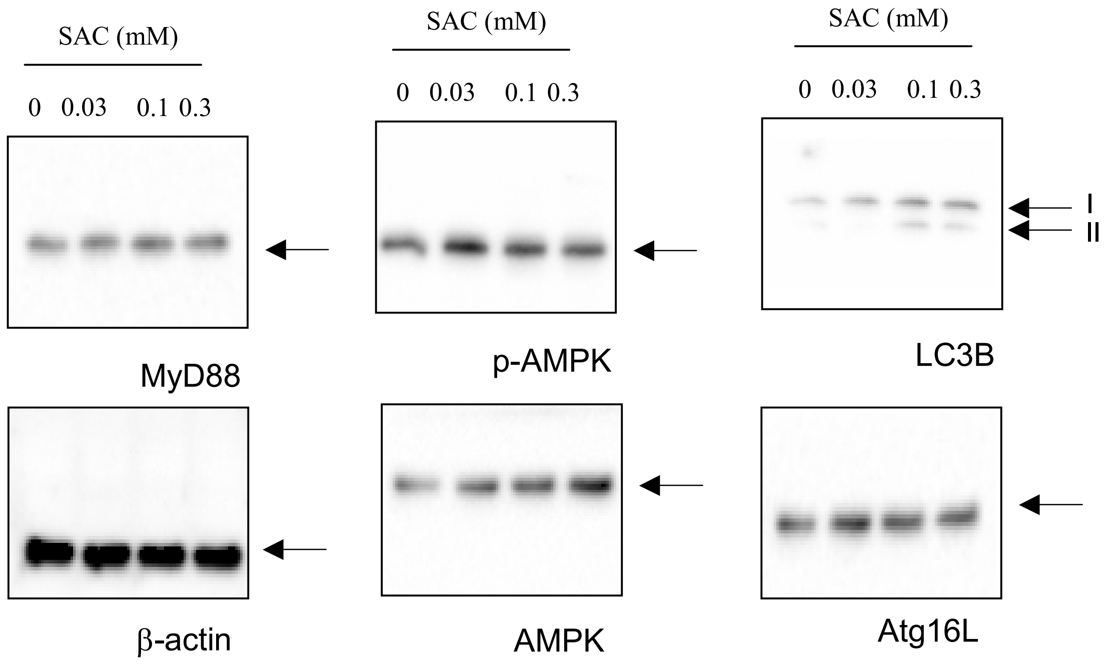

Fig. S5. Effect of SAC on LPS-induced IL-6 production and MyD88 degradation in splenic lymphocytes.

(a)The effect of SAC (0.03, 0.1, 0.3 and 1 mM) on IL-6 production induced by LPS (1 μg/ml) in splenic lymphocytes for 24 h was examined by ELISA. Data are shown as mean ± SD, n=4-5.

(b) The effects of SAC (0.03, 0.1, and 0.3 mM) on autophagy-related signaling pathway were examined by immunoblotting in splenic lymphocytes. Cell lysates were analyzed by western blotting with antibodies indicated.

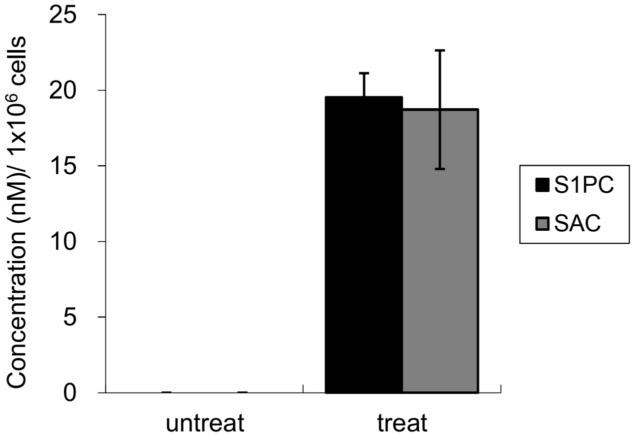

Fig. S6. Cellular uptake of S1PC and SAC into splenic lymphocytes.
Cellular uptake of S1PC (0.3 mM) and SAC (0.3 mM) for 5 min into splenic lymphocytes was examined by the mass spectrometer.
Data are shown as mean ± SD, n=4.

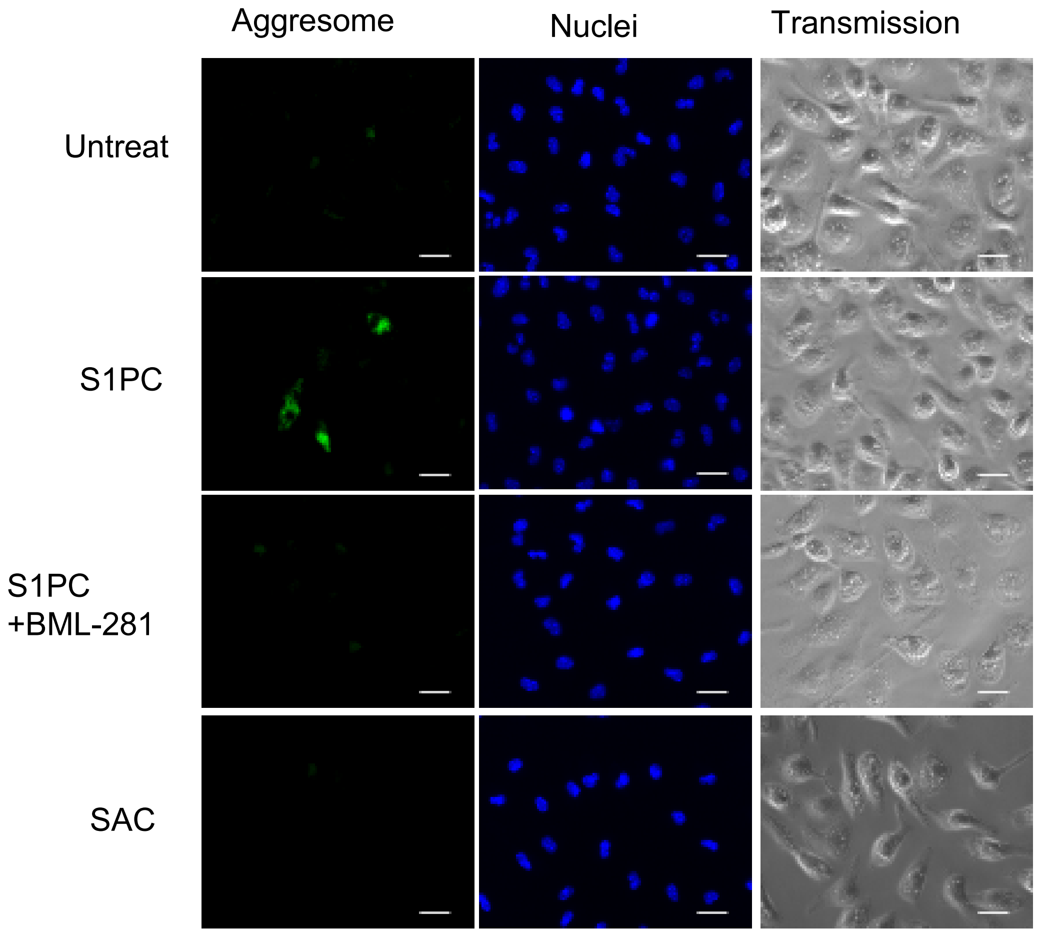

Fig. S7. Immunofluorescence imaging of aggresome formation

The effects of S1PC (0.3 mM) and SAC (0.3 mM) on aggresome formation with or without BML-281 (100 nM) in murine peritoneal macrophages for 10 min were examined by the aggresome detection kit and DAPI for nuclei. Bar: 20 μm.

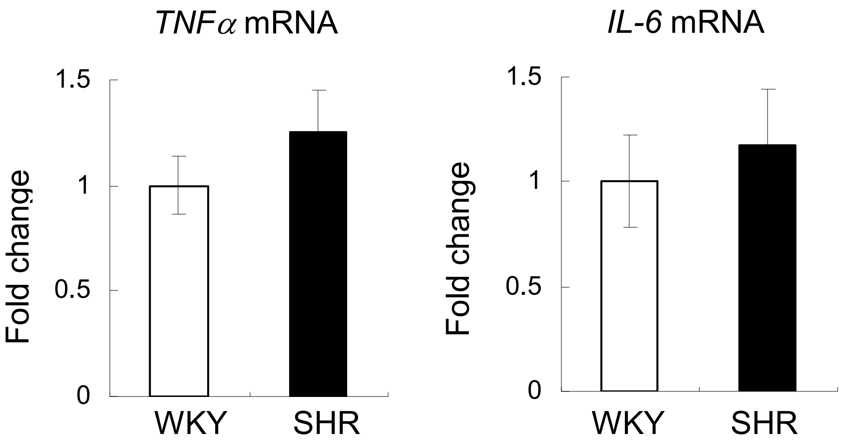

Fig. S8.

S1PC (6.5 mg/kg) was orally administrated to SHR for 10 weeks. The expression of TNFα and IL-6 mRNA in liver of SHR and WKY rats were examined by real-time PCR. Data are shown as mean ± SE, n=4-6.

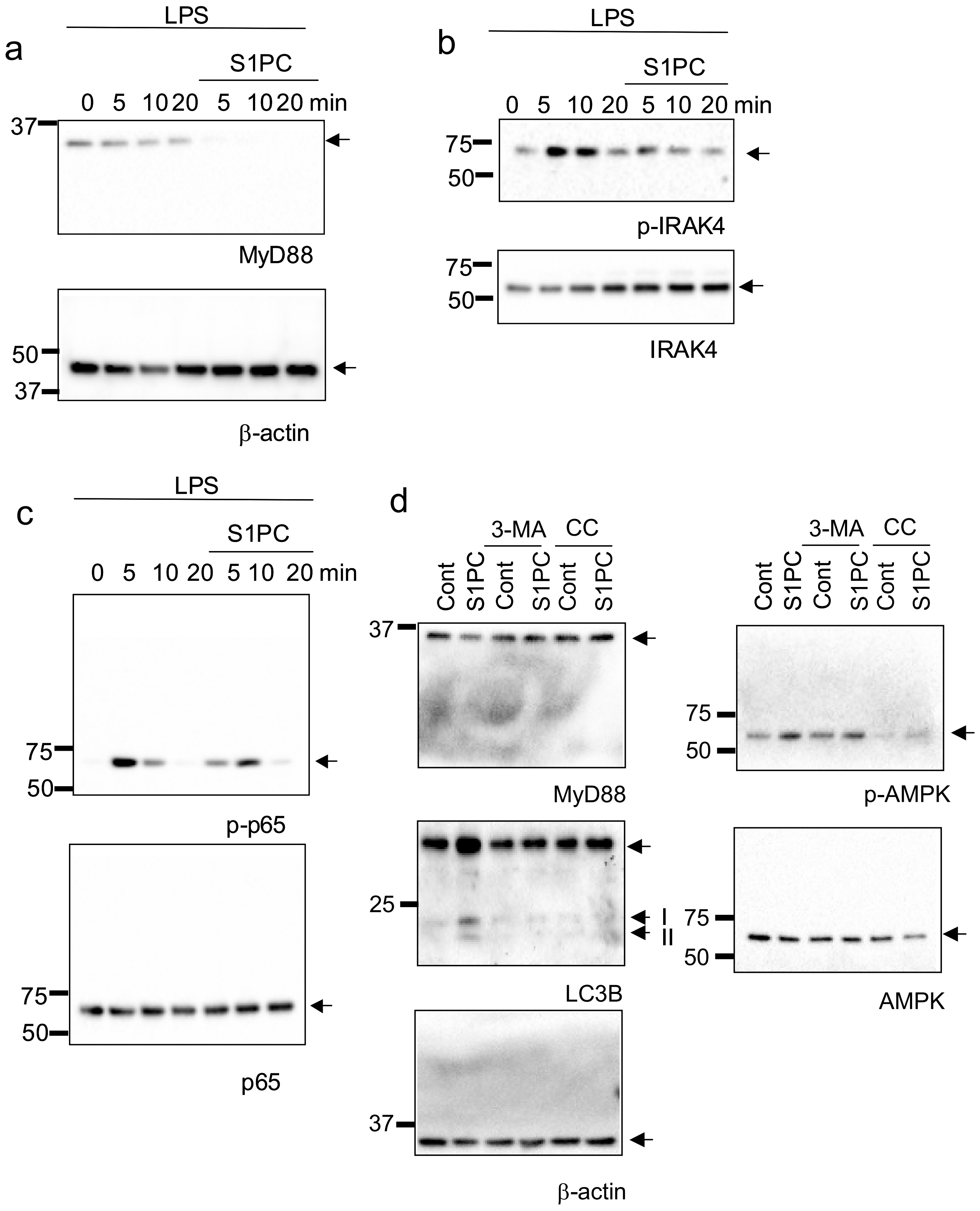

Fig. S9.

Full-width of membrane with a protein marker besides the original blots used in Figures 1c (a-c) and 1d (d). The molecular size is shown as indicated.

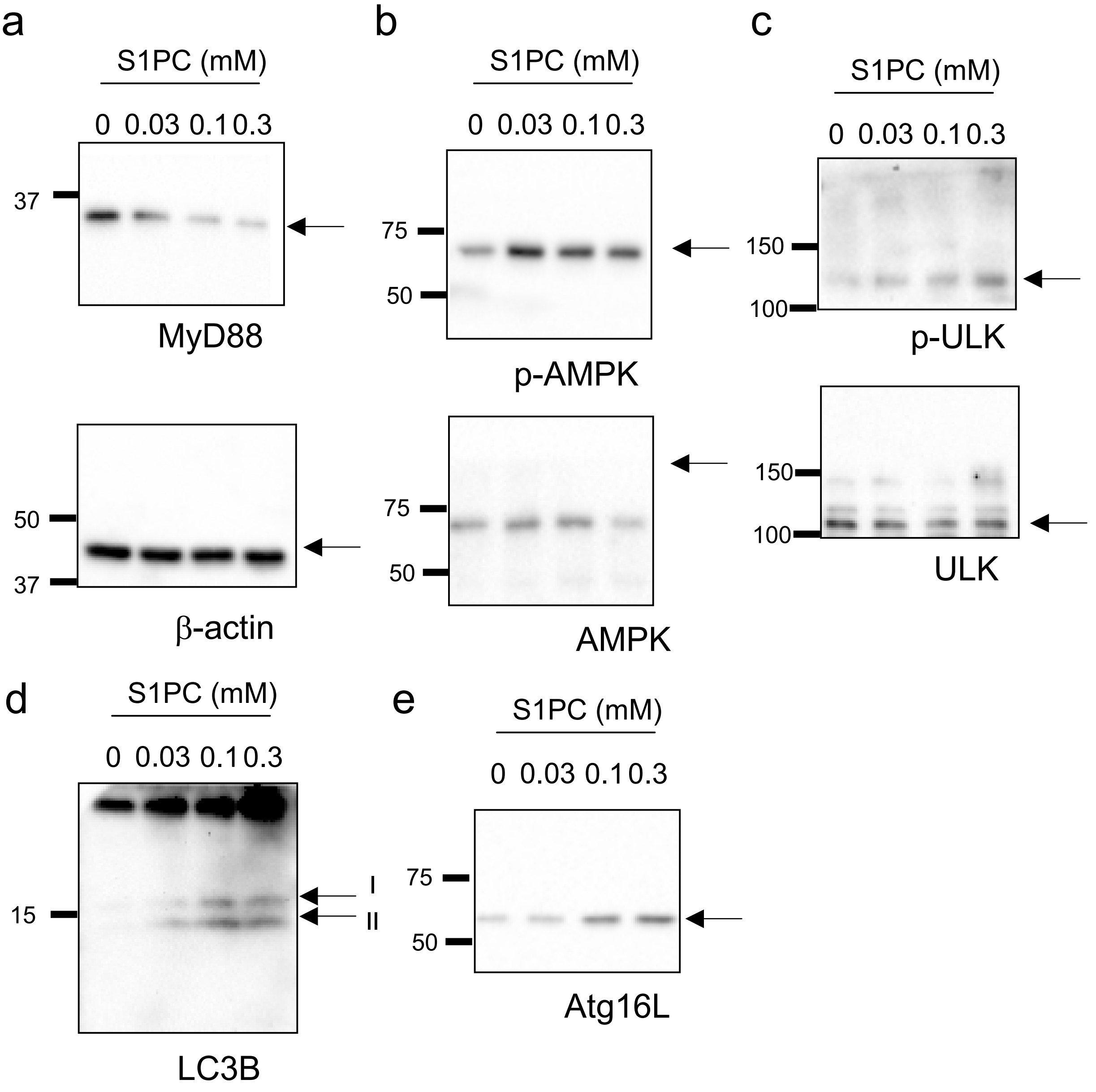

Fig. S10.

Full-width of membrane with a protein marker besides the original blots used in Figure 1e.

The molecular size is shown as indicated.

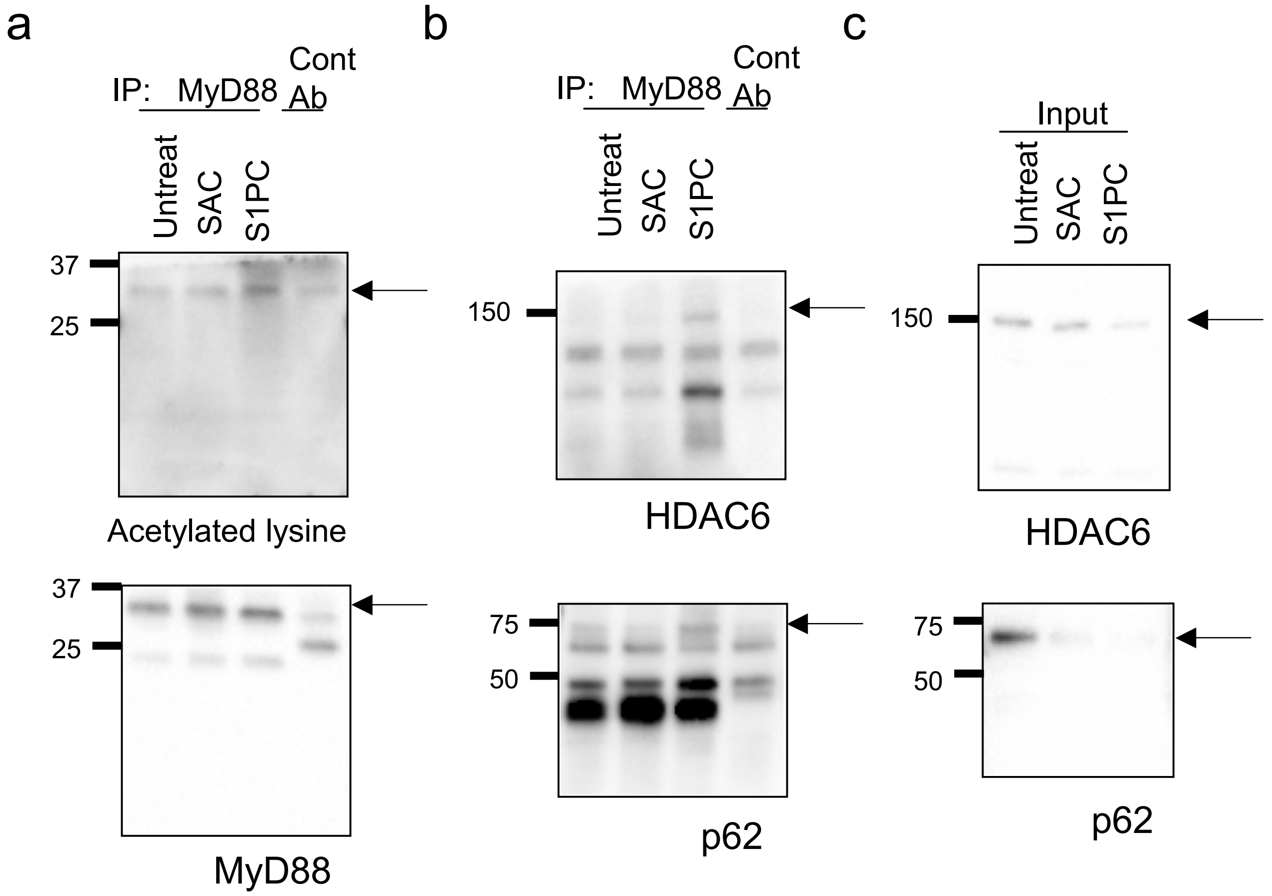

Fig. S11.

Full-width of membrane with a protein marker besides the original blots used in Figures 3c (a) and 3f (b and c). The molecular size is shown as indicated.

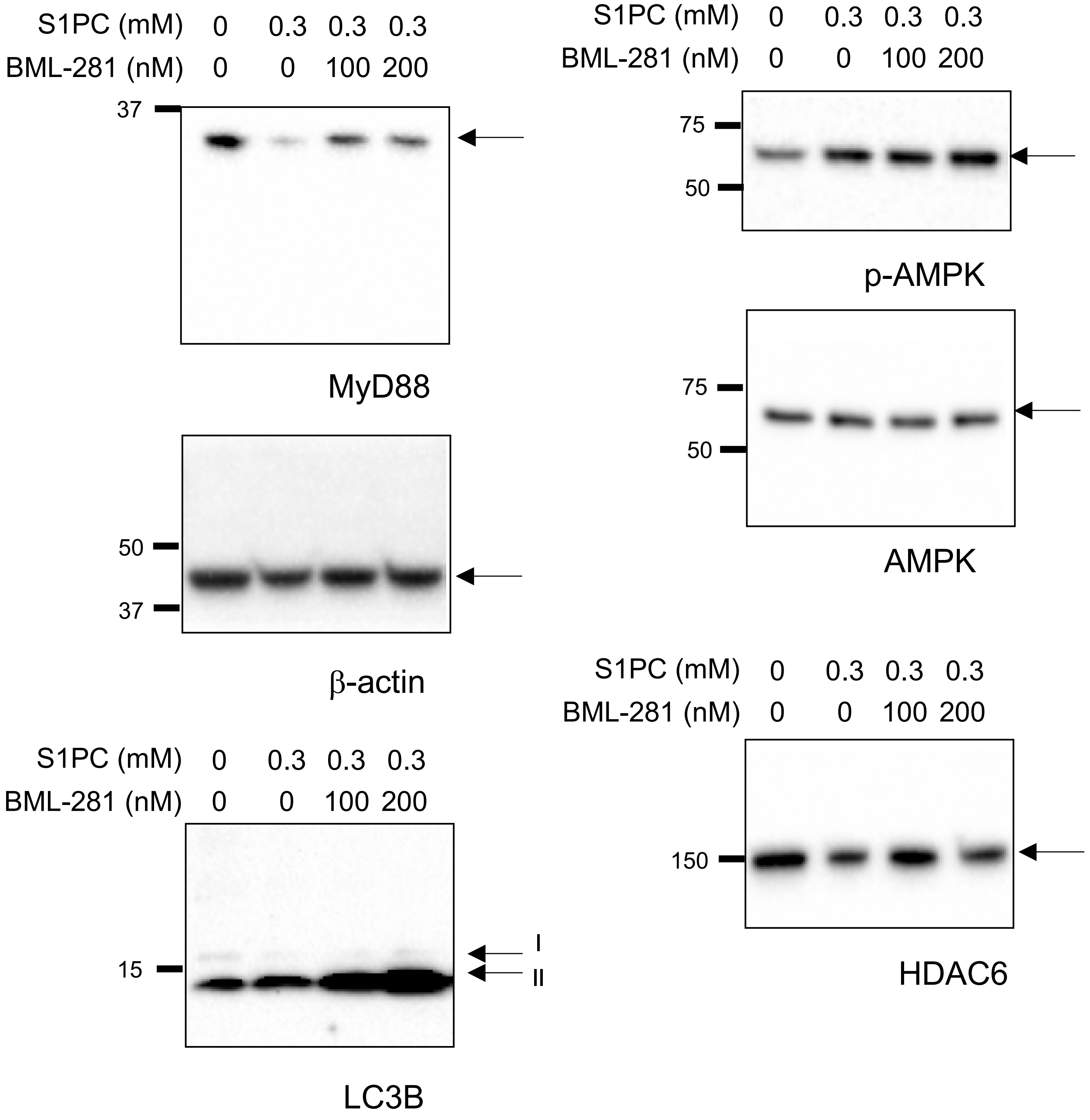

Fig. S12.

Full-width of membrane with a protein marker besides the original blots used in Figure 3g.

The molecular size is shown as indicated.

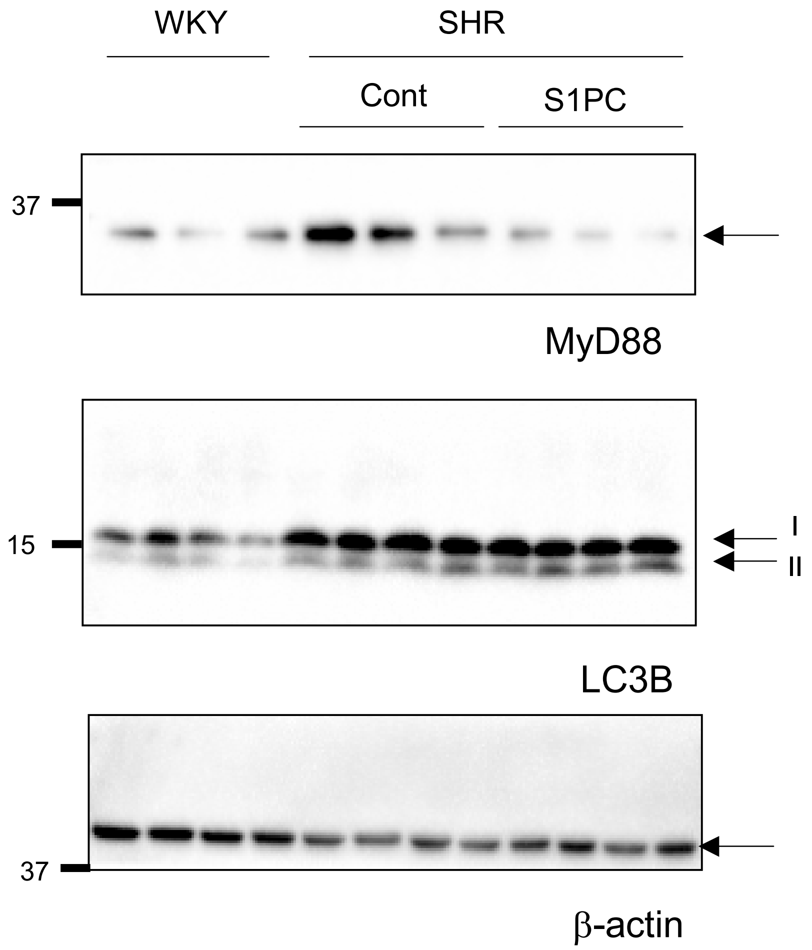

Fig. S13.

Full-width of membrane with a protein marker besides the original blots used in Figure 4

The molecular size is shown as indicated.
